# A Comprehensive Overview of Antibacterial Agents for Combating Multidrug-Resistant Bacteria: The Current Landscape, Development, Future Opportunities, and Challenges

**DOI:** 10.3390/antibiotics14030221

**Published:** 2025-02-21

**Authors:** Ina Gajic, Nina Tomic, Bojana Lukovic, Milos Jovicevic, Dusan Kekic, Milos Petrovic, Marko Jankovic, Anika Trudic, Dragana Mitic Culafic, Marina Milenkovic, Natasa Opavski

**Affiliations:** 1 Institute of Microbiology and Immunology, Faculty of Medicine, University of Belgrade, 11000 Belgrade, Serbia; milos.jovicevic@med.bg.ac.rs (M.J.); dusan_vk@yahoo.com (D.K.); jankovic.marko1987@gmail.com (M.J.); 2 Group for Biomedical Engineering and Nanobiotechnology, Institute of Technical Sciences of SASA, Kneza Mihaila 35/IV, 11000 Belgrade, Serbia; nina.tomic@itn.sanu.ac.rs; 3 Academy of Applied Studies Belgrade, College of Health Sciences, 11000 Belgrade, Serbia; bojanall@yahoo.com; 4 University Clinical Hospital Center “Dr. Dragisa Misovic-Dedinje”, 11040 Belgrade, Serbia; milos.m.petrovic@outlook.com; 5 Faculty of Medicine, University of Novi Sad, 21000 Novi Sad, Serbia; anika.trudic@mf.uns.ac.rs; 6 Institute for Pulmonary Diseases of Vojvodina, Sremska Kamenica, 21204 Novi Sad, Serbia; 7 Faculty of Biology, University of Belgrade, 11000 Belgrade, Serbia; mdragana@bio.bg.ac.rs; 8 Department of Microbiology and Immunology, Faculty of Pharmacy, University of Belgrade, 11000 Belgrade, Serbia; marina.milenkovic@pharmacy.bg.ac.rs

**Keywords:** antimicrobial resistance, multidrug resistance, new antibiotics, treatment options

## Abstract

**Background/Objectives:** Antimicrobial resistance poses a major public health challenge. The World Health Organization has identified 15 priority pathogens that require prompt development of new antibiotics. This review systematically evaluates the antibacterial resistance of the most significant bacterial pathogens, currently available treatment options, as well as complementary approaches for the management of infections caused by the most challenging multidrug-resistant (MDR) bacteria. For carbapenem-resistant Gram-negative bacteria, treatment options include combinations of beta-lactam antibiotics and beta-lactamase inhibitors, a novel siderophore cephalosporin, known as cefiderocol, as well as older antibiotics like polymixins and tigecycline. Treatment options for Gram-positive bacteria are vancomycin, daptomycin, linezolid, etc. Although the development of new antibiotics has stagnated, various agents with antibacterial properties are currently in clinical and preclinical trials. Non-antibiotic strategies encompass antibiotic potentiators, bacteriophage therapy, antivirulence therapeutics, antimicrobial peptides, antibacterial nanomaterials, host-directed therapy, vaccines, antibodies, plant-based products, repurposed drugs, as well as their combinations, including those used alongside antibiotics. Significant challenges exist in developing new antimicrobials, particularly related to scientific and technical issues, along with policy and economic factors. Currently, most of the alternative options are not part of routine treatment protocols. **Conclusions and Future Directions:** There is an urgent need to expedite the development of new strategies for treating infections caused by MDR bacteria. This requires a multidisciplinary approach that involves collaboration across research, healthcare, and regulatory bodies. Suggested approaches are crucial for addressing this challenge and should be backed by rational antibiotic use, enhanced infection control practices, and improved surveillance systems for emerging pathogens.

## 1. Introduction

**Figure 1:**
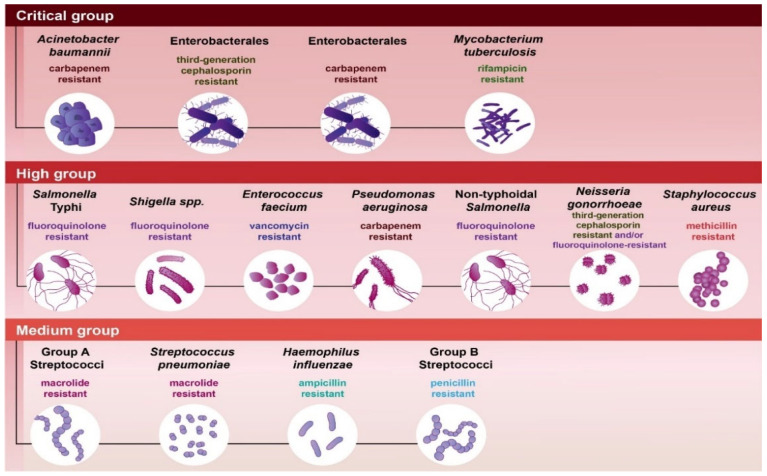
Bacterial priority pathogens list (based on: [2]).

The emerging threat of multidrug-resistant (MDR) bacteria poses one of the most significant challenges to global health. Infections caused by MDR pathogens can lead to the ineffectiveness of standard treatments, longer duration of illnesses, increased mortality, and higher healthcare costs. The alarming trend of the rise in antimicrobial resistance underscores the urgent need for a comprehensive evaluation of antibacterial agents that demonstrate efficacy against MDR strains. The most critical bacteria known for their ability to evade the effects of antibiotics are categorized under the acronym “ESKAPEE” (i.e., *Enterococcus faecium*, *Staphylococcus aureus*, *Klebsiella pneumoniae*, *Acinetobacter baumannii*, *Pseudomonas aeruginosa*, *Enterobacter species*, and *Escherichia coli*) [1]. Furthermore, the World Health Organization (WHO) has classified major bacterial pathogens into critical, high, and medium priority groups, highlighting the urgent need for new antibiotics and guiding research on novel antibacterial agents. These “priority” pathogens are a major health threat due to their disease burden, antibiotic resistance, and potential for severe infections (Figure 1).

In 2021, it was estimated that 4.71 million deaths were associated with bacterial antimicrobial resistance (AMR), including 1.14 million deaths attributable to bacterial AMR [3]. In 2020, over 800,000 infections in the European Union/European Economic Area (EU/EEA) were caused by MDR bacteria, leading to more than 35,000 deaths, according to data from the European Antimicrobial Resistance Surveillance Network [4]. Methicillin-resistant *Staphylococcus aureus* (MRSA) was estimated to cause more than 148,000 infections and 7000 deaths in 2015 [5,6]. In the United States of America (USA), the prevalence of healthcare-associated infections (HAIs) caused by MDR bacteria increased by 20% during the COVID-19 pandemic, peaking in 2021 [7]. In 2022, rates for all major MDR pathogens, except MRSA, remained above pre-pandemic levels [8]. In particular, MRSA and vancomycin-resistant *Enterococcus* (VRE) caused 323,700/10,600 and 54,500/5400 cases/deaths in 2019, respectively [9]. *Mycobacterium tuberculosis* was the second leading cause of death from a single infectious agent worldwide in 2022, with an estimated 1.3 million fatalities. The reported number of people newly diagnosed with tuberculosis (TB) was 7.5 million in 2022, which is the highest number since WHO began global TB monitoring. In 2019, almost half a million people developed rifampicin-resistant tuberculosis, undermining global efforts to control the disease [10].

In response to this urgent public health threat, various treatment strategies have been explored, including the reintroduction of older antibiotics and the development of new antimicrobial agents, including novel beta-lactam/beta-lactamase inhibitor (BLBLI) combinations. However, due to the limited treatment options, there is an urgent need for new approaches and agents with innovative mechanisms of action. Therefore, this article attempts to provide a thorough review of the latest treatment strategies used to combat various antibiotic-resistant bacteria that are currently responsible for severe healthcare-associated and community-acquired infections. Specifically, the objectives of this review were to: address the burden and impact of infections caused by the most relevant MDR bacteria, identify important bacterial resistance phenotypes, summarize current antibacterial treatment options, assess their efficacy and mechanisms of action, and explore ongoing advancements and future strategies for combating AMR.

## 2. Major Mechanisms of Action of Antibacterial Agents

**Figure 2:**
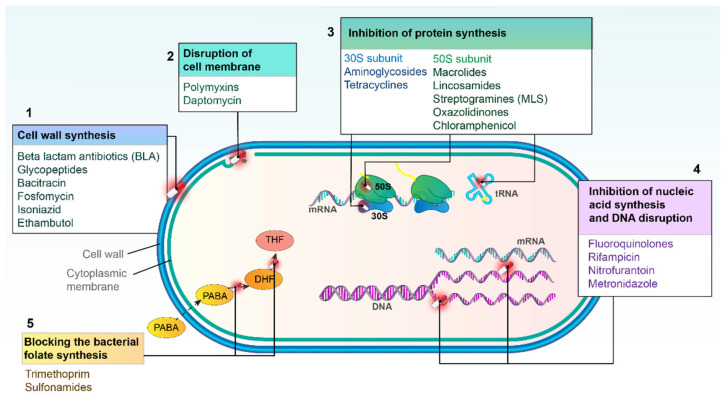
Molecular insights into the mechanisms of action across different antibiotic classes.

Antibiotics are chemical substances produced by microorganisms that inhibit the growth of or destroy bacteria. While antibiotics are natural products, semi-synthetic or fully synthetic (chemotherapeutics) agents are chemically engineered to enhance their effectiveness or broaden their spectrum of activity. Based on the mode of action, antibacterial agents can be classified into five groups as displayed in Figure 2.

Table S1 displays the key antibacterial agents with their spectrum of activity, and their corresponding classes.

## 3. Overview of Antibiotic Resistance Mechanisms

**Figure 3:**
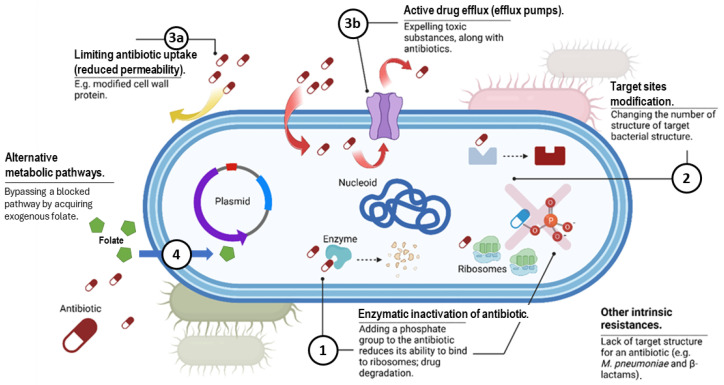
Summary of major antimicrobial resistance strategies. (1) Enzymatic drug inactivation; (2) modifying the drug target: transpeptidases, also known as penicillin-binding proteins (PBPs); ribosomes, and DNA gyrase and topoisomerase; (3) changes in drug transport (3a: limiting drug uptake; 3b: active drug efflux); and (4) utilizing alternative metabolic pathways.

Bacteria have evolved complex mechanisms to evade antimicrobial agents. Of note, bacteria can use multiple biochemical pathways to resist a single antibiotic. Major antimicrobial resistance strategies are depicted in Figure 3.

Antibiotic resistance mechanisms are generally classified as intrinsic or acquired. A natural mechanism observed in most species or intrinsic resistance can be constitutive, occurring independently of prior antibiotic exposure (e.g., lack of a cell wall or reduced permeability of the outer membrane), or induced by antibiotics or environmental stressors (e.g., activation of efflux pumps or biofilm formation) [11]. Acquired resistance arises from mutations or the acquisition of resistance genes, often following antimicrobial exposure [12]. Gram-negative bacteria employ all of these mechanisms, whereas Gram-positive bacteria are less likely to restrict drug uptake and lack certain drug efflux systems [13]. A crucial role in the emergence of MDR strains is the acquisition of mobile genetic elements, such as bacteriophages, plasmids, and integrative and conjugative elements [14] through conjugation, transduction, and transformation.

### 3.1. Production of Enzymes That Deactivate the Drug

The enzymatic breakdown and modification of antibiotics is a major and widespread bacterial resistance mechanism, especially in Gram-negative bacteria. This includes beta-lactamases that degrade beta-lactam antibiotics (BLAs), as well as enzymes that alter aminoglycosides, macrolides, and chloramphenicol. The genes for these enzymes are usually on plasmids, allowing easy transfer between bacteria through conjugation and consequent rapid resistance spread. The main mechanism of resistance to BLAs involves the destruction of these compounds by the action of beta-lactamases [15]. To date, over 8000 distinct beta-lactamases have been identified [16]. The classification of beta-lactamases based on their structure, along with the most significant enzymes, is summarized in Table 1.

Beta-lactamases are relatively rare in Gram-positive bacteria, but they are particularly prevalent in *Enterobacterales* and among Gram-negative non-fermenting bacteria. Beta-lactamase genes (*bla*) are the largest class of antibiotic resistance genes accounting for almost 50–70% of all known antibiotic resistance incidences [18].

Beta-lactamase inhibitors (BLIs) counteract beta-lactamase activity by protecting BLAs they are combined with. New beta-lactam and BLI combinations (BLBLIs) are discussed below.

#### 3.1.1. Beta-Lactamases

Class A beta-lactamases include narrow spectrum enzymes, extended-spectrum beta-lactamases (ESBLs) and carbapenemases. Genes encoding these enzymes are highly diverse and can be classified into several families, such as TEM, SHV, IRT, CMT, CTX-M, GES, PER, VEB, BEL, TLA, SFO, and OXY [19]. A CTX-M-type beta-lactamases emerged as the most common ESBLs globally, surpassing TEM and SHV as the dominant type.

Class C beta-lactamases or AmpC enzymes often remain undetected by routine testing, leading to misdiagnosis and inappropriate antibiotic treatment. Although AmpC enzymes are typically chromosomally encoded, plasmid-mediated variants have also been identified [20]. However, a new stage in the development of resistance was identified with the emergence of extended-spectrum beta-lactamases AmpC (ESAC), a subset of AmpC enzymes that, like ESBLs, efficiently hydrolyze penicillins, cephamycins, and third-generation cephalosporins but are inactive against carbapenems. Unlike ESBLs, ESACs are not inhibited by conventional BLIs. When *Enterobacterales* combine AmpC or ESAC production with porin loss, carbapenem resistance can occur.

Class D beta-lactamases, also known as OXA-type enzymes, are not always classified as ESBLs. Some OXA-type enzymes are also capable of hydrolyzing carbapenems, making them a significant concern in treating MDR infections. In *Acinetobacter baumannii*, carbapenem resistance is predominantly mediated by oxacillinases, including intrinsic OXA-51 and acquired OXA variants (e.g., OXA-23, OXA-24/40, OXA-58, OXA-143, and OXA-235). While OXAs weakly hydrolyze carbapenems, resistance is enhanced when *bla_OXA_* genes are overexpressed via mobile insertion elements, such as IS*Aba1*.

Class B beta-lactamases present a major challenge in combating antibiotic resistance because they can degrade a broad range of BLAs, including carbapenems. However, these carbapenemases are typically ineffective against monobactams and are a major driver of carbapenem resistance. Prominent MBL genes, such as IMP, VIM, and NDM, can be either chromosomally encoded or plasmid-mediated, enabling their transfer between bacteria through horizontal gene transfer [21]. Indeed, numerous reports highlight the global dissemination of carbapenem-resistant *A. baumannii* (CRAB) strains, carriers of the MBLs [22,23]. In 2022, NDM emerged as the most common carbapenemase in carbapenem-resistant *Klebsiella pneumoniae* (CRKP) across Europe [24]. The emergence and rise of carbapenem-resistant *P. aeruginosa* (CRPA) have been mostly attributed to the production of MBLs (VIM, IMP, NDM) [25]. Of note, plasmids carrying the *bla_NDM_* gene frequently harbor additional resistance genes conferring resistance to various beta-lactamases, quinolones, and aminoglycosides, further complicating treatment [26].

#### 3.1.2. Enzymes That Alter Antibiotics

Aminoglycoside-modifying enzymes (AMEs) catalyze drug modification leading to reduce aminoglycoside binding to ribosomal targets, making them a major resistance mechanism [27]. Macrolide detoxification involves two enzyme classes: macrolide phosphotransferases and macrolide esterases. The high-level resistance of certain bacteria to chloramphenicol is due to the enzyme chloramphenicol acetyltransferase, which modifies the antibiotic to a biologically inactive derivative.

### 3.2. Modification of the Target Site of the Drug

Even minor changes in molecules essential for bacterial survival can hinder the antibiotics’ ability to bind to them, resulting in the development of antibiotic resistance.

Thus, the production of new penicillin-binding proteins (PBPs), such as PBP2a and PBP2c, plays a key role in the development of methicillin-resistant staphylococci (MRS) [28]. In methicillin-resistant *S. aureus* (MRSA), the *mecA* and *mecC* genes encode PBPs with low affinity for BLAs, including penicillins, cephalosporins, and carbapenems. However, MRSA remains susceptible to ceftaroline, a fifth-generation cephalosporin designed to target PBP2a. Point mutations in PBPs are a significant mechanism of antibiotic resistance in various bacterial species (e.g., Group B Streptococci, *Streptococcus pneumoniae*, *Neisseria gonorrhoeae*).

Cell wall modification, specifically the replacement of peptidoglycan precursors with d-Ala-d-Lac or d-Ala-d-Ser, to which vancomycin has low affinity, leads to vancomycin resistance, usually resulting from the acquisition of *van* genes, which alters the vancomycin target site.

Bacteria have developed resistance to antibiotics that target ribosomal subunits and interfere with protein synthesis (e.g., macrolides, chloramphenicol, aminoglycosides, tetracyclines) through mutations in ribosomal genes [29]. Resistance to linezolid has emerged due to G22576T 23 rDNA gene mutations and/or the acquisition of resistance genes (*cfr*, *optrA*, *poxtA*). Cfr is a methyltransferase that catalyzes the post-transcriptional methylation of nucleotides in the 23S rRNA gene [30]. OptrA and PoxtA proteins are members of the ATP-binding cassette (ABC) protein superfamily that displace antibiotics from the ribosome. In a recent study, Fukuda et al. found that the *optrA*, *poxtA*, and *cfr* genes detected in various enterococci species derived from livestock compost may serve as reservoirs of linezolid transferable resistance genes [31]. It is important to point out that linezolid resistance genes and mutations can be detected in phenotypically linezolid-susceptible isolates. These strains may promote spread and trigger a rapid development of linezolid resistance, potentially leading to treatment failure.

Mutations in the genes encoding DNA gyrase and topoisomerase lead to high-level resistance to fluoroquinolones, which is one of the most common mechanisms of resistance to these antibiotics [32]. In *Enterobacterales*, quinolone resistance typically arises from mutations in the quinolone resistance-determining region (QRDR) of genes encoding the DNA gyrase subunits (GyrA, GyrB) and topoisomerase IV (ParC, ParE), particularly in *gyrA* and *parC*. Additionally, plasmid-mediated quinolone resistance (PMQR) mechanisms have been identified, such as Qnr, which protects the target enzymes, AAC(6′)-Ib-cr, which mediates acetylation of certain quinolones, and efflux pumps like QepA and OqxAB.

Lipid A modification, driven by plasmid-mediated mobile colistin resistance (*mcr*) genes and mutations in chromosomal genes involved in lipid A biosynthesis, is the primary mechanism of colistin resistance. The ability of *mcr* genes to transfer easily to susceptible strains is particularly concerning, as it facilitates the spread of resistance.

Daptomycin resistance involves alterations of the cell surface, where increased positive charge causes electrostatic repulsion of the daptomycin–calcium complex. In *S. aureus*, mutations in the multiple peptide resistance factors (mprF) lead to higher lysinylation of peptidoglycan, increasing cell-surface charge and preventing the attachment of the calcium—daptomycin complex [33]. Additionally, reduced expression of negatively charged membrane phospholipids also changes the membrane charge and leads to daptomycin resistance in *S. aureus* [34]. It has been found that increased teichoic acid synthesis in the cell wall and the degree of its D-alanylation can play a role in some daptomycin-resistant strains. Interestingly, the mechanism of resistance to daptomycin appears to differ among the two species of enterococci. A recent discovery revealed a new mutation in the *cls* gene that causes daptomycin resistance in *E. faecium* [35]. In *E. faecalis*, daptomycin resistance is due to mutations in the LiaFSR signaling systems, which control cell membrane and cell wall homeostasis. This causes cardiolipin redistribution from the septum to non-septal locations on the cell membrane, preventing daptomycin oligomerization in the septal cell membrane [36]. Previous genomic studies indicated that full expression of daptomycin resistance requires additional mutations in enzymes glycerophosphoryl diester phosphodiesterase and cardiolipin synthase [37].

### 3.3. Changes in Antibiotic Transport, Including Decreased Permeability and Increased Efflux

Antibiotic transport alteration can occur due to reduced influx and increased efflux. Decreased permeability results from mutations in the genes encoding porins, which alter the size, hydrophobicity, or charge of these channels. This modification prevents the diffusion of hydrophilic drugs and leads to resistance [38]. This phenomenon is common among Gram-negative bacteria and is particularly significant in *P. aeruginosa* resistance to BLAs and other antibiotics.

Efflux pumps, located in the cytoplasmic membrane of bacteria, are essential for regulating solute levels and contribute to antibiotic resistance by expelling drugs from the cells [39]. Importantly, efflux systems have been implicated in conferring resistance to almost all classes of antibiotics.

### 3.4. Alteration of Metabolic Pathways

Metabolic pathway alteration contributes to antibiotic resistance through the (1) overproduction of folate-synthesizing enzymes, which “overwhelm” the ability of trimethoprim/sulfamethoxazole (TMP/SMX) to inhibit folate production, or (2) utilization of external folate sources, such as tetrahydrofolic and folinic acid, as seen in enterococci [40].

## 4. Antibiotic Resistance in Key Gram-Negative Bacteria and Available Treatment Options

Currently, carbapenem-resistant enterobacteria (CRE), CRAB, and ESBL-positive enterobacteria represent the most significant challenges in the treatment of healthcare-associated infections.

### 4.1. Carbapenem-Resistant Acinetobacter baumannii

In the past few decades, *A. baumannii* has rapidly emerged as a significant cause of morbidity and mortality in healthcare settings. Carbapenems were once the primary treatment for nosocomial infections caused by *A. baumannii*. However, the widespread emergence of CRAB strains, mainly driven by OXA enzymes and, to a lesser extent, MBLs, presents major treatment challenges due to the ease with which these resistance mechanisms transfer [22,23,41]. Apart from beta-lactamases, carbapenem resistance in *A. baumannii* is additionally ascribed to nonenzymatic mechanisms, mainly including loss or disruptions of outer membrane proteins (CarO, OmpA, Omp33, OprB, Omp25, OprC, OprD, OmpW, and *dacD) *and high expression of multidrug efflux pumps (high expression of resistance-nodulation-division (RND) family-type AdeABC pump) [42].

Polymyxins and tigecycline are one of the few remaining antimicrobial options for CRAB. However, *A. baumannii* resistance to colistin, a last-resort antibiotic, has been reported globally due to the complete loss or modification of lipopolysaccharide (LPS) [43]. Although still scarce, clinical tigecycline-resistant *A. baumannii* isolates were reported from different geographic regions, and the main resistance mechanisms were overexpression of efflux pumps, altered outer membrane permeability, altered tigecycline targets, production of tigecycline-inactivating enzymes, and activation of repair pathways mediating tigecycline resistance after DNA damage [44]. Currently, for pandrug-resistant *A. baumannii*, limited therapeutic options exist; these include a novel siderophore cephalosporin, aztreonam-avibactam, and combination therapies of two or more antibiotics or novel approaches such as bacteriophage therapy or prophylactic vaccination [45,46]. Of note, Zosurabalpin, an antibiotic that targets the LPS transport machine, belonging to a new class, is now in phase 1 clinical trials [47,48].

### 4.2. Carbapenem-Resistant Pseudomonas aeruginosa

Carbapenem resistance in *P. aeruginosa* is multifactorial and includes the acquisition of carbapenemase-encoding genes through horizontal gene transfer, deficiency or repression of the carbapenem porin (OprD), overexpression of the mexAB-oprM efflux pump and overexpression of the chromosomal gene encoding the *P. aeruginosa* intrinsic cephalosporinase (*ampC*). As discussed above, the emergence of carbapenemase-producing *P. aeruginosa* has been mostly attributed to the production of MBLs and, to a lesser extent, class A beta-lactamases (e.g., KPC and GES) [25].

The increasing rate of infections caused by MDR, extensively drug-resistant (XDR), and particularly CRPA has led to the resurgence of colistin as a critical last-resort therapeutic option. Similar to CRPA, the increased use of colistin has led to the global emergence of *P. aeruginosa* strains with reduced susceptibility to this antibiotic, with the highest rates of colistin resistance exceeding 10% in certain regions of Asia and Africa [49].

Meanwhile, the approved BLBLI combinations, ceftazidime/avibactam, ceftolozane/tazobactam, meropenem/vaborbactam, and imipenem/cilastatin/relebactam have demonstrated in vitro effectiveness in treating CRPA. However, emerging resistance and cross-resistance to all listed agents has been reported. Moreover, cefiderocol, a siderophore cephalosporin, was recently developed as a single antibiotic without a BLI to tackle all carbapenem resistance mechanisms, including the production of MBLs. It has shown potent activity and proven efficacy in clinical studies, particularly among patients with life-threatening infections [25].

### 4.3. Third-Generation Cephalosporin-Resistant Enterobacterales

Resistance to third-generation cephalosporins typically arises from the production of ESBLs and plasmid-mediated AmpC beta-lactamases, as described in Section 3.1.1. The presence of third-generation cephalosporin-resistant *Enterobacterales* greatly restricts treatment options. Although carbapenems may still be effective, rising resistance to these antibiotics presents further challenges.

### 4.4. Carbapenem-Resistant Enterobacterales

The growing resistance of *Enterobacterales* to third-generation cephalosporins has resulted in increased use of carbapenems, leading to a rise in CRE prevalence. Three major mechanisms are responsible for carbapenem resistance: the production of enzymes that hydrolyze carbapenem antibiotics, efflux of antibiotics, and the loss or mutation of porins [50]. The production of carbapenemases, such as MBLs and certain serine β-lactamases (e.g., KPC, OXA-48-like enzymes) is the most critical mechanism due to the high potential for horizontal gene transfer, which facilitates the rapid spread of these resistant strains among various bacterial species highlighting the need for robust surveillance and novel therapeutic strategies. *Enterobacterales* can produce AmpC or ESBL, which, when combined with alterations in porin synthesis, may lead to carbapenem resistance [51].

Major carbapenemases and efficacies of novel BLBLIs against CRE are shown in Table 1. Currently, BLBLIs have been recommended as the first-choice treatment for class A-producing CRE. Notably, carbapenem resistance in Gram-negative bacilli has led to the resurgence of polymyxins as a last-line treatment. However, the emergence of colistin resistance among CREs poses a significant challenge. Other treatment options are tigecycline, plasomycin, and fosfomycin.

### 4.5. Fluoroquinolone-Resistant Salmonella Typhi

Fluoroquinolone-resistant *Salmonella* Typhi is a major public health issue, especially in areas where typhoid fever is endemic.

In addition to the mechanisms of fluoroquinolone resistance involving mutations in the genes encoding DNA gyrase and topoisomerase IV, modifications in efflux pumps, porins, and plasmid-mediated quinolone resistance (*qnr* genes) have also been identified [52]. In regions with high resistance rates, such as certain African and Asian countries, fluoroquinolones have become less effective, prompting increased use of alternatives like third-generation cephalosporins and azithromycin. However, resistance to these drugs is steadily increasing, highlighting the need for continued surveillance and research.

### 4.6. Fluoroquinolone-Resistant Non-Typhoidal Salmonella

Fluoroquinolone-resistant non-typhoidal *Salmonella* (NTS), such as *Salmonella* Enteritidis and *Salmonella* Typhimurium, has emerged as a significant public health challenge worldwide, causing foodborne and waterborne illnesses. The presence of fluoroquinolone-resistant NTS is particularly concerning as it restricts treatment options for severe cases, such as septicemia. In such instances, alternative antibiotics, such as third-generation cephalosporins or azithromycin, may be used, but rising resistance to these drugs poses additional challenges.

### 4.7. Fluoroquinolone-Resistant Shigella spp.

The emergence of fluoroquinolone-resistant *Shigella* complicates the management of severe cases of bacillary dysentery, as ciprofloxacin is currently recommended by the WHO as the primary treatment for dysentery patients resistant to third-generation cephalosporins and nalidixic acid. Quinolone resistance is caused by changes in the activity of efflux pumps and specific genetic mutations in the QRDR of DNA gyrase and DNA topoisomerase [53].

### 4.8. Resistance in Neisseria Gonorrhoeae

Ceftriaxone is the last remaining option for empiric first-line monotherapy for gonorrhea. In 2019, The European committee on antimicrobial susceptibility testing (EUCAST) updated the clinical resistance breakpoint for azithromycin from minimum inhibitory concentration (MIC) > 0.5 mg/L to an epidemiological cut-off (ECOFF) of MIC > 1 mg/L [54]. Despite this change, there was a significant increase in the proportion of isolates exceeding the azithromycin ECOFF from 7.6% in 2018 to 25.6% in 2022. The proportion of isolates showing resistance to ciprofloxacin also increased from 57.7% in 2020 to 65.9% in 2022. Due to the rising resistance of *N. gonorrhoeae* to azithromycin and ciprofloxacin, neither antibiotic is recommended for monotherapy unless susceptibility is confirmed [55].

Although dual ceftriaxone and azithromycin resistance is exceedingly rare in Europe, the rapidly decreasing azithromycin susceptibility combined with the continued detection of occasional ceftriaxone resistance threatens the effectiveness of treatment and control of gonorrhea [56,57]. Due to this, the European treatment guideline was updated in 2020 to recommend high-dose ceftriaxone plus azithromycin dual therapy or ceftriaxone high-dose monotherapy, which is now most frequently used [58]. Even though the prevalence of resistance to cefixime has significantly decreased from 1.4% in 2018 to 0.3% in 2021, cefixime resistance needs to be monitored closely, as gonococcal strains with resistance to both cefixime and ceftriaxone continue to spread globally [57,59].

In recent years, several Phase II and III trials for newer antimicrobials (zoliflodacin, gepotidacin, delafloxacin, and solithromycin) have been carried out with promising results [60]. In 2023, zoliflodacin met its primary endpoint in a global pivotal phase III clinical trial. Study investigators found that oral zoliflodacin demonstrated statistical non-inferiority of microbiological cure when compared to treatment with intramuscular injection of ceftriaxone and oral azithromycin, a current global standard of care regimen. Zoliflodacin was found to be generally well tolerated and there were no serious adverse events or deaths recorded in the trial. It inhibits type II topoisomerase, essential for bacterial function and reproduction. Previous in vitro studies have shown that it is active against MDR strains of *N. gonorrhoeae*, including those resistant to ceftriaxone and azithromycin, with no cross-resistance with other antibiotics [61]. Recently, the outer membrane vesicle (OMV) meningococcal B vaccine, MeNZB, was reported to be associated with reduced rates of gonorrhoea following a mass vaccination campaign in New Zealand [62]. There is a high level of sequence identity between MeNZB OMV and Bexsero OMV antigens, and between the antigens and gonococcal proteins. Bexsero induces antibodies in humans that recognize gonococcal proteins [63]. These results provide a proof of principle for prospective gonococcal vaccine development.

### 4.9. Resistance in Helicobacter pylori

In 2017, clarithromycin-resistant *Helicobacter pylori* was listed as a WHO priority pathogen. However, in 2024, it was removed from the list despite rising resistance, based on new evidence and expert opinion [2]. Since 1998, clarithromycin resistance has risen, leading to decreased treatment success [64,65]. Resistance to alternative antibiotics like levofloxacin and metronidazole has also increased, reaching over 15% and 40% in some regions, respectively [66,67]. As a result, the standard triple therapy with clarithromycin or levofloxacin was changed to quadruple therapy with or without bismuth or sequential therapy [68,69]. Currently, rifasutenizol, a rifamycin-nitroimidazole conjugate, is in Phase 3 trials, showing promise for *H. pylori* treatment with dual-action mechanisms, particularly conceived and developed to treat microaerophilic and anaerobic bacterial infections. In a recent report, its monotherapy and combination therapy were presented as well-tolerated, with a suggested triple regimen as an efficient option for *H. pylori* therapy [70]. Additionally, SVT-1C469, a probiotic mix, is being explored to replace harmful bacteria and modulate immune responses to inhibit *H. pylori* growth [71]. A two-stage Phase 1 trial evaluating the safety and efficacy of this live bacterial therapy for treating *H. pylori* infection is registered by the Australian New Zealand Clinical Trials Registry (ACTRN12620000923965) [72]. While the trial is completed, no public peer-reviewed data have been released yet.

## 5. Antibiotic Resistance in Important Gram-Positive Cocci and Available Treatment Options

### 5.1. Methicillin-Resistant Staphylococcus aureus

**Figure 4:**
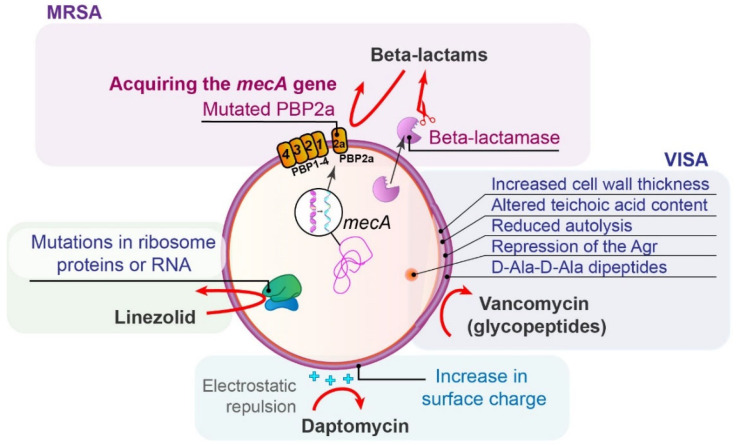
Resistance of *Staphylococcus aureus* to beta-lactam antibiotics, vancomycin, linezolid, and daptomycin. VISA, vancomycin-intermediate *S. aureus*; MRSA, methicillin-resistant *S. aureus*.

*S. aureus* can acquire resistance to BLAs through two main mechanisms: the production of penicillinases (class A according to Ambler) and modifications to PBPs (Figure 4). In MRSA strains, the resistance primarily occurs through the alteration of PBPs, as described in Section 3.2. [73].

Initially, healthcare-associated MRSA (HA-MRSA) was the dominant form. Over time, however, it has increasingly appeared as community-acquired MRSA (CA-MRSA). Of note, not all antibiotic-resistant bacteria exhibit resistance in standard tests (heterogeneous resistance), so the most reliable method to confirm resistance is by detecting the *mecA* or *mecC* genes, encoding altered PBPs [74].

Treatment options for MRSA include vancomycin, daptomycin, linezolid, tigecycline, ceftaroline, clindamycin, TMP/SMX, and fosfomycin, with vancomycin being a key option.

Vancomycin-non-susceptible *S. aureus* (e.g., vancomycin-resistant *S. aureus*—VRSA, vancomycin-intermediate *S. aureus*—VISA, and heterogeneous vancomycin-intermediate *S. aureus—*hVISA) are rare but emerging phenotypes. Nine operons (*vanA-E*, *vanG*, *vanL-N*) confer glycopeptide resistance, with the *vanA* genes producing D-Ala-D-Lac, which has a much lower affinity to vancomycin compared to the wild-type D-Ala-D-Ala [6]. This resistance often arises from the transfer of the *vanA* gene from VRE [6,75]. Of note, VISA strains lack *van* determinants and show increased cell wall thickness, altered teichoic acid content, reduced autolysis, and repression of the Agr, staphylococcal global regulator. Mutations in regulatory genes result in thicker, poorly cross-linked cell walls that block antibiotic entry. Decoy D-Ala-D-Ala dipeptides on the surface further hinder vancomycin access, becoming clogged with the drug [76]. Increased expression of PBP2 and PBP2a “traps“ vancomycin, while reduced PBP4 expression and cross-linking contribute to VISA phenotype. hVISA is thought to precede the development of VISA [77]. Additionally, VISA strains show some daptomycin resistance due to their thicker and more positively charged cell walls.

The emergence of a daptomycin-resistant mutant of *S. aureus* during enduring therapy is a common finding and is described in Section 3.2.

Resistance of *S. aureus* to linezolid is mediated by multiple mechanisms: through mutations of the L3, L4, and L22 ribosomal proteins or a mutation of the 23S ribosomal RNA, G2576T [78].

### 5.2. Vancomycin-Resistant Enterococci

VRE accounts for more than 20% of enterococcal infections, with *E. faecium* particularly exhibiting high-level resistance to multiple antibiotics. Plenty of plasmid-encoded *van* genes that encode enzymes that modify the D-Ala-D-Ala precursor have been found in VRE. In *Enterococcus* spp. *vanA* and *vanB* are the most frequently detected resistance determinants conferring high-level resistance to vancomycin/teicoplanin, or only vancomycin, respectively. The VanA phenotype is manifested by inducible high-level resistance to vancomycin and teicoplanin. VanB phenotypes exhibit inducible resistance to vancomycin, but remain susceptible to teicoplanin [79].

Treatment options for VRE include: linezolid, daptomycin, tigecycline, quinupristin/dalfopristin, ampicillin, and ceftaroline. The prevalence of resistance to linezolid is steadily increasing in Europe and has been widely detected both in clinical and non-clinical settings [80]. Many data sources indicate that daptomycin resistance in *E. faecium* is due to electrostatic repulsion of the daptomycin–calcium complex from the cell membrane, as described in detail in Section 3.2.

### 5.3. Macrolide-Resistant Streptococcus pneumoniae

In treating pneumococcal diseases, BLAs, and macrolides are first choice antibiotics. In *S. pneumoniae*, macrolide resistance arises primarily from dimethylation of ribosomal 23S rRNA by an enzyme encoded by *erm*B to prevent antibiotic binding, as well as from efflux via a two-component pump encoded by the *mef*E or *mef*A (macrolide efflux) genes [81].

Ribosomal methylation by *ermB* confers resistance to macrolides, lincosamides, and streptogramin B, which is characterized as the MLS_B_ phenotype and provides high-level resistance to macrolides. On the contrary, *S. pneumoniae* isolates that exhibit efflux pumps are classified as having the M-phenotype with low level of resistance to 14- and 15-membered macrolides only. The relative prevalence of the two macrolide resistance mechanisms differs by geographic region. In most European countries, about 90% of *S. pneumoniae* isolates exhibit the MLS_B_ phenotype [82,83], while in North America, 50% to 65% of resistant *S. pneumoniae* isolates have efflux mutations, which are associated with lower levels of macrolide resistance [84]. Macrolide-resistant *S. pneumoniae* often exhibit resistance to penicillin as well, posing a significant therapeutic challenge. In pneumococci, modifications to cell wall PBPs lead to a gradual decrease in affinity for penicillin [85].

### 5.4. Macrolide-Resistant Group A Streptococci

The first-line antibiotics in the treatment of Group A streptococci (GAS) infections are penicillins and macrolides. Macrolide-resistant GAS (MRGAS) has increased, while GAS isolates remain sensitive to BLAs. Similar to *S. pneumoniae*, resistance to macrolides in GAS arises from ribosomal methylation coded by *erm*A and *erm*B genes and expressed as iMLS and cMLS phenotype, respectively, as well as more prevalent antibiotic repulsion via efflux pumps, encoded by *mef*A gene (M phenotype). In penicillin-hypersensitive patients with infections caused by MRGAS, treatment options include clindamycin, fluoroquinolones, vancomycin, linezolid, or certain cephalosporins for patients with mild penicillin allergy.

### 5.5. Penicillin-Resistant Group B Streptococci

The resistance level of Group B streptococci (GBS) to penicillin is categorized as low to medium according to the WHO, meaning that the prevalence ranged from 5% to 10% [2]. Additionally, the prevalence of penicillin-resistant GBS is on the rise. Resistance rates, however, differ by region; in Europe and the United States of America, penicillin-resistant GBS remains exceptionally rare, whereas Japan has reported a higher prevalence of 6% [86]. The MICs for penicillin non-susceptible GBS are near the susceptible breakpoint, meaning that misclassification of these isolates is not infrequent due to the inherent imprecision of antimicrobial susceptibility testing, even by reference methods. Accordingly, the Clinical and laboratory standards institute guidelines do not suggest routinely testing strains for nonsusceptibility to penicillin. Moreover, there is a recommendation to reassess the penicillin breakpoints or to explore the use of alternative BLA for more accurate detection of non-susceptibility [86]. Although reports of GBS with reduced susceptibility or penicillin MIC values above the ECOFF have been reported sporadically around the world since 1994, the first molecular characterization of these strains was reported in Japan in 2008 [87]. Point mutations in *pbp2x* are primarily found to be responsible for this phenotype, even though, several other point mutations have been reported both in *pbp1a*, *pbp2a*, and *pbp2b* [88]. These strains are frequently also resistant to erythromycin, clindamycin, tetracycline, and fluoroquinolones [89].

Erythromycin, clindamycin, and vancomycin are second-line treatment options for patients with severe penicillin allergy or those with infections caused by penicillin-non-susceptible GBS. High and increasing rates of clindamycin resistance in GBS [90] mean that empirical use of clindamycin cannot be relied upon for the prevention or treatment of GBS in patients non-eligible for penicillin therapy, and it should only be used when the strain is known to be susceptible. In cases of penicillin resistance, alternative options include: clindamycin, vancomycin, linezolid, third-generation cephalosporins, or daptomycin in severe cases. For neonatal infections caused by penicillin-resistant GBS, vancomycin is often the first-line option, sometimes in combination with gentamicin.

### 5.6. Ampicillin-Resistant Haemophilus Influenzae

Before routine vaccination, *H. influenzae* type B (Hib) was the commonest cause of invasive *H. influenzae* disease, occurring predominantly in healthy children younger than five years, for whom it was the commonest cause of bacterial meningitis. The introduction of the Hib conjugate vaccine into national immunization programs in the early 1990s led to a rapid and sustained reduction in Hib disease across all age groups [91]. This, in turn, opened up space for an increase in invasive non-typeable cases of *H. influenzae* (NTHi), as well as invasive non-b-serotype cases [92,93].

Ampicillin is the first-line treatment for *H. influenzae* infections; however, global resistance to the drug is steadily increasing. The primary mechanism of ampicillin resistance in *H. influenzae* is the production of β-lactamase (e.g., *bla_TEM-_1* gene, *bla_ROB-1_*). Strains resistant to ampicillin via β-lactamase production are classified as β-lactamase-positive, ampicillin-resistant (BLPAR). The second mechanism involves amino acid substitutions in the transpeptidase enzyme PBP3, encoded by the *ftsI* gene.

Strains that do not produce β-lactamase but are non-susceptible to ampicillin are classified as β-lactamase-negative, ampicillin-resistant (BLNAR). Strains with both β-lactamase production and PBP3 alteration are classified as β-lactamase-positive, amoxicillin-clavulanate-resistant (BLPACR). Both BLNAR and BLPACR strains are resistant to numerous BLAs, including penicillins and cephalosporins [94]. The global prevalence of β-lactamase-producing *H. influenzae* and MDR *H. influenzae* has been reported to be 34.9% and 23.1%, respectively. The prevalence of MDR *H. influenzae* is notably higher in Asian countries (24.6%) compared to Western regions (15.7%) [95].

### 5.7. Rifampicin-Resistant Mycobacterium tuberculosis

**Figure 5:**
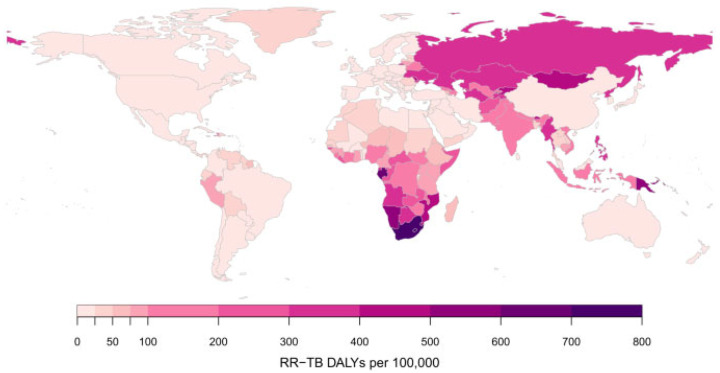
Estimated DALYs per 100,000 due to incident RR-TB in 2020 by country [96]. DALYs, disability-adjusted life years.

Drug-resistant tuberculosis (DR-TB) continues to be a public health threat. The WHO uses five categories to classify cases of DR-TB: isoniazid-resistant TB; rifampicin-resistant TB (RR-TB); multidrug-resistant TB (MDR-TB); extensively drug-resistant TB (XDR-TB); and pre-XDR-TB. MDR-TB is TB resistant to rifampicin and isoniazid. Pre-XDR-TB is TB resistant to rifampicin and any fluoroquinolone (a class of second-line anti-TB drugs), while XDR-TB is TB resistant to rifampicin, plus any fluoroquinolone, plus at least one of either bedaquiline or linezolid. Since rifampicin is the most effective first-line drug, both MDR-TB and RR-TB require treatment with second-line drugs [94]. Per population, RR-TB disability-adjusted life years (DALYs) were highest in the former Soviet Union and southern African countries, as shown in Figure 5.

Since 2018, WHO has recommended all-oral regimens for the treatment of MDR/RR-TB, marking a major advance compared with previous regimens that included injectable agents [97]. Treatment success rates in 2022 were 88% for people treated for drug-susceptible TB and 63% for people with MDR/RR-TB [98]. The latest guidelines for the treatment of DR-TB, updated in 2022, include three major categories of regimens. The first is a short 6-month all-oral regimen for people with MDR/RR-TB (which may be extended by 3 months if necessary) consisting of bedaquiline, pretomanid, linezolid, and moxifloxacin; for people with pre-XDR-TB, the regimen can be used without moxifloxacin. The second is all-oral short regimens of 9 months for people with MDR/RR-TB (which may be extended by 2 months if necessary). The third category is longer regimens of 18–20 months that may include an injectable drug (amikacin). The short 6-month regimen is prioritized for use and is recommended for people aged 14 years and older who have MDR/RR-TB or pre-XDR-TB [99]. As of August 2023, there were 28 drugs for the treatment of TB disease in Phase I, Phase II, or Phase III trials. The 28 drugs comprise: 18 new chemical entities [BVL-GSK098, BTZ-043, delpazolid, GSK-286 (GSK 2556286), GSK-3036656, macozinone, OPC-167832, TBAJ-587, TBAJ-876, TBI-223, TBI-166, TBA-7371, telacebec-(Q203), sanfetrinem, SQ109, SPR720 (fobrepodacin), sutezolid, and sudapyridine (WX-081)], three drugs that have already been approved by WHO for use in treatment [bedaquiline, delamanid and pretomanid], and seven repurposed drugs [clofazimine, levofloxacin, linezolid, moxifloxacin, rifampicin (high dose), rifapentine and tedizolid] [98].

### 5.8. Resistance in Clostridioides Difficile

*Clostridioides difficile* infection (CDI) is a leading cause of antibiotic-associated diarrhea and a major public health concern [100,101]. Current first-line therapies include vancomycin and fidaxomicin for initial episodes and recurrences, while metronidazole is now limited to non-severe cases of CDI. Resistance to all proposed antibiotics has been reported [102]. The last Food and Drug Administration (FDA)-approved antibiotic for CDI was fidaxomicin in 2011, while five new antibiotics are currently in clinical trials [103]: (1) Ridinilazole—an oral non-absorbable antibiotic that disrupts DNA binding, promoting cell death in *C. difficile*. It reduces CDI recurrence and maintains microbiome diversity without increasing resistance [104]; (2) CRS3123 (REP 3123)—also an oral antibiotic and a selective inhibitor of methionyl-tRNA synthetase that shows narrower spectrum activity than vancomycin and fidaxomicin, while benefiting the microbiome [105]; (3) Oxaquin—an intravenous oxazolidinone-quinolone hybrid that spares Gram-negative components of the gut microbiome [106]; (4) Ibezapolstat—a dichlorobenzyl purine analogue that inhibits bacterial DNA polymerase IIIC, demonstrating good tolerance, high stool concentrations, and sparing of important gut microbiota [107]; (5) MGB-BP-3—an oral minor groove binder that inhibits the growth of Gram-positive bacteria through DNA interference. It shows potential as a new first-line treatment for CDI with promising safety and efficacy reports [108].

## 6. Overview of Existing Antibiotics Effective Against Particular MDR Strains

### 6.1. Beta-Lactam Antibiotics/Inhibitors of Beta-Lactamases

**Figure 6:**
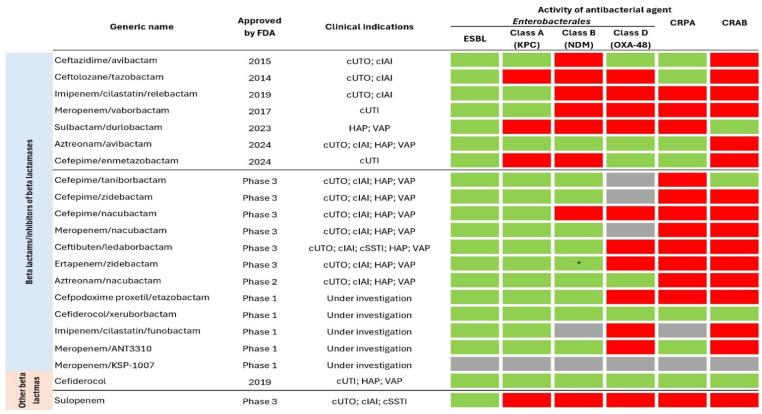
Newly approved and in-trial beta-lactams alone or combined with beta-lactamase inhibitors, with clinical indications and spectrum of activity against ESBL—positive *Enterobacterales*, and carbapenem-resistant *Enterobacterales*, *Pseudomonas aeruginosa*, and *Acinetobacter baumannii*. Green, active; red, inactive; grey, insufficient data, cUTI; complicated urinary tract infections, cIAI; complicated intraabdominal infections; HAP, hospital-acquired pneumonia; VAP, ventilator-associated pneumonia; cSSTI, complicated skin and soft tissue infections; CRPA, carbapenem-resistant *Pseudomonas aeruginosa*; CRAB, carbapenem-resistant *Acinetobacter baumannii*; * Only *E. coli*.

The BLBLI antibiotics have been utilized in clinical practice for over 40 years, starting with the introduction of amoxicillin/clavulanic acid. In the past decade, several new BLBLI have been introduced, including ceftazidime/avibactam, ceftolozane/tazobactam, imipenem/relebactam, and meropenem/vaborbactam. Clinical indications of BLBLI and their activity against carbapenem-resistant Gram-negative bacilli are presented in Figure 6. They are all last-resort antibiotics for treating infections caused by MDR Gram-negative bacteria; however, mechanisms of resistance have been identified for each of them.

#### Rare Genotypes of Gram-Negative Bacilli Resistant to New BLBLI

Variants of KPC (e.g., KPC-31, KPC-35, KPC-41, KPC-50, and KPC-53) can confer resistance to ceftazidime/avibactam (CAZ/AVI). Additionally, mutations in genes-encoding ESBLs, such as CTX-M15 and CTX-M14 along with OXA-48, and changes in porins Omp35 and Omp36, which reduce membrane permeability, can also contribute to resistance [109].

Ceftolozane/tazobactam works synergistically, with ceftolozane inhibiting PBPs and tazobactam targeting serine beta-lactamases. Although ceftolozane/tazobactam is less affected by porin permeability variations or efflux pumps, *Pseudomonas*-derived cephalosporinase (chromosomally encoded class C cephalosporinase) is capable of hydrolyzing ceftolozane [110,111].

Resistance to imipenem/relebactam may be driven by mutations in the porins Omp35 and Omp36, increased expression of efflux pumps, increased AmpC expression, and production of ESBLs like SHV and TEM-1 [109].

Described mechanisms of resistance to meropenem/vaborbactam include decreased expression of the porins Omp35 and Omp36 in KPC-3-producing strains of *K. pneumoniae*. Along with reduced membrane permeability, increased expression of efflux pumps, particularly AcrAB-TolC, may also contribute to resistance against this antibiotic [109].

Sulbactam/durlobactam, a narrow-spectrum antibiotic that is effective against CRAB [112,113]. Sulbactam, a penicillanic acid sulfone beta-lactam, was introduced as a BLI and has been widely used in combination with ampicillin. Its inhibitory activity primarily targets class A beta-lactamases, while its antibacterial efficacy against *Acinetobacter* spp. is attributed to its action on PBP1 and PBP3 [112].

Durlobactam (ETX-2514), on the other hand, is a novel modified diazabicyclooctane (DBO) beta-lactamase inhibitor, with a broader spectrum of activity against class A, C, and D beta-lactamases compared to avibactam [113]. However, same as avibactam, it is not effective against MBL. Durlobactam also exhibits antibacterial activity against certain *Enterobacterales* by binding to and inhibiting PBP2 [114]. It enters *A. baumannii* cells through the outer membrane porin OmpA [115]. Regarding resistance mechanisms, alterations or deletions in OmpA are not considered significant resistance mechanisms [116]. In vitro studies have shown that mutations targeting PBP3, the target of sulbactam, as well as the activity of efflux pumps, may contribute to resistance [116,117].

Enmetazobactam differs from tazobactam by having a single methyl group, promoting bacterial wall penetration. Cefepime-enmetazobactam is active against class A, C, and D beta-lactamases [118]. However, it is more effective against ESBL producers than carbapenemase-producing *Enterobacterales*. In *P. aeruginosa*, enmetazobactam did not enhance the potency of cefepime [119].

Aztreonam, a well-known monobactam, has limited use due to the rise of ESBL and AmpC-type beta-lactamases, in recent years. MBLs can hydrolyze all BLA except aztreonam. Since MBLs are often combined with ESBLs, it showed limited results, with the activity against 30% of strains [120]. Avibactam, a non-beta-lactam BLI, can inhibit many beta-lactamases including ESBLs and AmpC. The success of the aztreonam-avibactam (ATM/AVI) combination in treating MBL-producing bacteria was inferred from the therapeutic effectiveness observed with the aztreonam and ceftazidime/avibactam combination [121]. Currently, ATM/AVI-resistant strains are rare, but some mechanisms of resistance have been identified. For example, an *E. coli* strain with the *bla_CMY-141_* gene and PBP3 mutation has shown resistance to this antibiotic. Additionally, *Enterobacter hormaechei* strains harboring the *bla_CTX−M-15_* gene, exhibiting overexpressed act-17 and modified OmpC and PBP3, have demonstrated resistance to all BLBLIs, including ATM/AVI. *K. pneumoniae* strains with KPC-2 and VEB-31 variants also exhibited resistance to ATM/AVI [122].

### 6.2. New Non-BLBLI Antibiotics Effective Against MDR Strains

Cefiderocol is a newly developed intravenous synthetic conjugate consisting of a cephalosporin component and a catecholin-type siderophore. It can bind extracellular iron and then use its active transport system in bacteria to enter the cells. Once inside the bacteria, it releases the cephalosporin active compound that reacts with PBP3, blocking the cell wall synthesis. It has a broad spectrum of activity against a variety of MDR bacteria carrying all beta-lactamase classes (Figure 6), or porin loss/efflux pumps overexpression [123]. While the drug demonstrated strong effectiveness against MBL-positive isolates, the rapid development of bacterial resistance, even during treatment is discouraging [124,125]. Additionally, the presence of heteroresistance among Gram-negative bacilli is reported, though its clinical significance remains unclear [126]. Potential mechanisms include the interplay between changes in the expression and/or mutation in the iron transportation system, production of beta-lactamases, and mutations in PBP3 [127].

Table 2 lists other new non-BLBLI antibiotics effective against MDR bacteria.

Eravacycline is a new intravenous and oral synthetic fluorocycline antibiotic, similar to tigecycline. The main mechanisms of resistance among tetracyclines, the efflux pumps and ribosomal protection proteins do not interfere with eravacycline as with tigecycline [135]. The resistance is detected among MDR bacteria: among CRKP, it is mediated by mutations in the gene encoding the Lon protease (an ATP-dependent protease, that participates in biofilm formation, motility, pathogenicity, and stress responses), upregulation of the multidrug efflux system AcrA-AcrB-TolC and porin proteins OmpA and OmpU [136,137]; in *A. baumannii*, it is mediated by overexpression of the efflux pump genes adeABC [138]. Nevertheless, the combination of eravacycline with ATM/AVI or CAZ/AVI displayed synergistic therapeutic effects [137].

Omadacycline [139] is a semi-synthetic aminomethylcycline analog of minocycline. It shows greater activity against MDR bacteria which use efflux pumps and ribosome protection as a resistance mechanism than older tetracyclines [140]. The property is limited not only to tetracycline resistance but also to quinolones, macrolides, and aminoglycosides [128].

Plazomicin [129] has a unique semisynthetic structure that lacks hydroxyl groups typical for classical aminoglycosides enabling it to overcome the main resistance mechanisms of aminoglycoside-inactivating enzymes [141]. Also, the beneficial side of its usage is reduced toxicity, in contrast to classical aminoglycosides due to the reversible type of nephrotoxicity, and importantly, it is non-ototoxic [142,143].

Lefamulin is the first pleuromutilin antibiotic used beyond the topical form in humans. Its mechanism of action is unique, inhibiting protein synthesis by preventing tRNA binding for peptide transfer of the 50S ribosome. Therefore, there is no cross-resistance with other antibacterial agents with similar mechanisms of action. Also, there is a low tendency for resistance development with a low risk for *C. difficile* development [144]. Also, in the last decade, a few new fluoroquinolone derivatives were introduced in clinical practice: delafloxacin, lascufloxacin, and levonadifloxacin **[130,145]**.

## 7. Combination Antibiotic Therapy Against MDR Bacteria

During the last two decades, antimicrobial agents have been increasingly used in combinations to provide a broad-spectrum activity, or to delay and inhibit the emergence of resistant bacterial strains [131,146]. Indications for combination antimicrobial therapy include: (a) empirical treatment of life-threatening infections; (b) polymicrobial infections; (c) prevention of the emergence of antimicrobial resistance; (d) more efficient killing of the pathogen; (e) lowering the dose of a single drug to reduce toxicity; and finally (f) interrupting multiple pathogenic mechanisms [146,147].

One of the most important reasons for combinational therapy is the emergence of MDR Gram-negative bacteria with very limited therapeutic options. Initially, the combination of meropenem and high-dose prolonged infusion of doripenem was adjusted for carbapenemase-producing *K. pneumoniae* with high carbapenem MIC values. In 2011. double carbapenem therapy (DCT) combining ertapenem and doripenem was proposed [148]. This was later expanded to combinations of ertapenem with doripenem or meropenem after successful treatments of patients with bacteremia and UTIs caused by pan-drug-resistant *K. pneumoniae* [149]. A systematic review found that DCT (ertapenem and meropenem or doripenem) offered similar efficacy compared to other regimens (colistin, tigecycline, aminoglycoside monotherapies, or their combination regimens) but significantly reduced mortality in CRE infections [150].

In patients with MBL-producing *Enterobacterales* infections, primarily for NDM-positive strains, a combination of ceftazidime/avibactam with the addition of aztreonam is preferred and shown to be more effective with a lower mortality rate compared to cefiderocol, colistin or tigecycline-based regime therapy [132,151].

In cases of difficult-to-treat *P. aeruginosa* without beta-lactam susceptibility (ceftolozane/tazobactam, ceftazidime/avibactam, imipenem/cilastatin/relebactam, or cefiderocol), the BLA with the lowest MIC can be combined with tobramycin if sensitive; polymyxin B, preferably over colistin, could be an alternative to tobramycin [133]. For CRAB, a regimen of sulbactam/durlobactam in combination with a carbapenem (imipenem/cilastatin or meropenem) is recommended and an alternative combination should be high-dose ampicillin-sulbactam with polymyxin B, minocycline, tigecycline or cefiderocol [134,152].

Ciprofloxacin may show greater effectiveness against biofilm-forming microorganisms and MDR isolates when combined with a variety of antibacterial agents, such as antibiotics from various classes, nanoparticles, natural products, bacteriophages, and photodynamic therapy [153].

The interaction of daptomycin with other antimicrobial drugs can be a promising therapy because, compared with other antimicrobial agents, daptomycin has a different mechanism of action and thus there is no cross-resistance with other antibiotics [154]. The combination of daptomycin with BLA offers a promising therapeutic option for complicated invasive MRSA infections, but further studies are needed to elucidate the mechanisms and to determine the in vivo efficacy of this combination [155]. Rand et al. showed the synergy of daptomycin with oxacillin and other BLAs against MRSA [156]. Lefebvre et al. examined the combination of daptomycin and rifampicin compared with the combination of vancomycin and rifampicin in the acute osteomyelitis model. Both combinations were more effective than the corresponding monotherapy [157].

Zhou et al. investigated the interaction between polycationic antimicrobial peptides (AMPs) with clinically used antimicrobial agents. The results of this study demonstrated synergy between ranalexin and polymyxin E, doxycycline, and clarithromycin [158].

## 8. Alternative Therapeutic Approaches and Repurposed Drugs

It is evident that even in developed countries, we are facing an overuse of antibiotics and consequently the emergence of MDR bacteria [159]. Therefore, scientists are focusing on researching and developing new molecules, natural products, and other innovative approaches to combat infections more effectively and sustainably [160,161].

### 8.1. Bacteriophage Therapy

Bacterial viruses or bacteriophages (phages) have long been recognized for treating bacterial infections but initially struggled to gain traction, particularly in the West. However, renewed interest in phage biology has emerged due to the growing threat of AMR, advancements in phage engineering, and awareness of the abundance of phages in all bacteria-rich environments, with genome sequencing revealing phage DNA within bacterial genomes. Phages offer a relatively safe usage profile for humans and can be quickly modified to combat emerging bacterial infections [162], gaining recognition in personalized medicine as a promising alternative for treating infections caused by difficult-to-treat bacteria.

**Figure 7:**
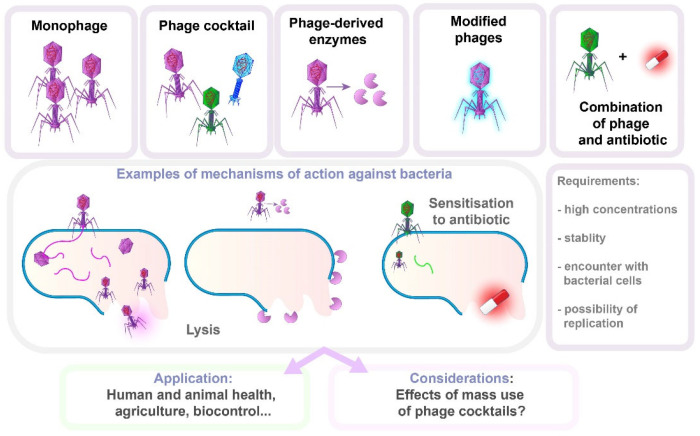
Application of bacteriophage cocktails alone or in combination with antibiotics in the treatment of bacterial infections.

A key feature of phage therapy is its high specificity, targeting specific bacterial strains or species. Thus, bacterial genotyping is crucial for selecting the right phage, aided by advances in sequencing technologies. The narrow spectrum of phage activity results in minimal disruption to the normal microbiota. However, many infections involve bacterial communities or biofilms, where multiple bacterial species or strains coexist. A single phage may not be effective against all the bacteria present, requiring a cocktail of phages, which complicates therapy. When combined with antibiotics, phage therapy may improve therapeutic outcomes, potentially offering a solution to the growing problem of AMR (Figure 7) [23,163].

The use of phages bears with it certain caveats: the inability to predict the effects of mass use of phage cocktails, especially when considering their application in animal health control and biocontrol. Also, according to some authors, the cost of phage treatment is likely to be high [164]. Additionally, for successful therapy, phages must be lytic, present in high concentrations, stable, unrestricted in encountering bacteria, and able to replicate. Importantly, bacteria may develop phage resistance [165]. The most important resistance mechanisms are receptor modification or loss, CRISPR-Cas systems, restriction-modification (R-M) systems, biofilm formation, and efflux pumps. The R-M system serves as a primary defense mechanism, protecting bacteria against foreign DNA that may originate from phages. This system relies on the presence of a restriction endonuclease that recognizes and cleaves the phosphodiester bond at restriction sites within the double-stranded foreign DNA, and a methyltransferase that catalyzes the addition of a methyl group to the host’s DNA. Methylation of recognition sites in the host genome prevents restriction, thereby allowing the R-M system to differentiate between self and foreign DNA. This defense system is not effective against single-stranded DNA or RNA molecules [166]. Another mechanism of bacterial adaptive immunity against foreign DNA (viruses and plasmids) is CRISPR-Cas systems, consisting of clustered regularly interspaced short palindromic repeats (CRISPR), associated with Cas proteins. In the adaptation phase, when the bacterium recognizes foreign DNA, Cas proteins cut fragments of this DNA, which are known as “spacers”. These spacers are then integrated into the CRISPR locus of the bacterial genome, creating a memory of previous infections. During the expression phase, this region is transcribed into a long RNA transcript or pre-CRISPR RNA (pre-crRNA), which is then processed into shorter crRNA, which serves as markers for newly encountered DNA. Finally, in the interference phase, when the bacterium is attacked again by the same virus or plasmid, the crRNA, in a complex with Cas proteins, recognizes and binds to the complementary sequence within the invading DNA. The Cas proteins then cut the foreign DNA, leading to its degradation [167,168,169].

Currently, there is no fully FDA-approved phage therapy in the United States, but there are approved clinical trials and “compassionate use” cases, especially for patients with antibiotic-resistant infections who lack other treatment options. While phage therapy has traditionally been advanced through numerous investigator-initiated trials since its discovery, a rising number of industry-sponsored trials now reflect increasing private-sector investment and commercial interest in the field [170].

### 8.2. Antivirulence Therapeutics

Antivirulence therapies represent a new and rapidly growing approach to treating bacterial infections, focusing on suppressing bacterial virulence rather than directly killing bacterial cells. This approach, known as antivirulence therapy (“pathoblockers”), inhibits pathogen virulence factors, thereby preventing bacteria from causing disease [160]. Key targets of these therapies include the bacterial type III secretion system (T3SS), quorum sensing, liposomes, etc. The advantages of antivirulence therapies are multiple: (i) reduced selective pressure compared to traditional antibiotics, which decreases the risk of resistance development; (ii) preservation of commensal microbiota, which plays an important role in protecting against secondary infections; (iii) avoidance of bacterial SOS responses, which are associated with severe side effects commonly seen with antibiotic use. These therapies have already shown potential for development into effective treatments for bacterial infections, either as standalone options or in combination with antibiotics, making them a promising tool in the fight against antibiotic resistance [171]. This particularly applies to plant products, where it has been shown that many of them can inhibit bacterial growth at different stages of their life cycle with an almost complete lack of side effects [172,173]. Also, the research of nanomaterials as antimicrobial agents is very promising since they could be featured with multiple antimicrobial mechanisms, which is challenging for antimicrobial resistance development [174].

### 8.3. Antimicrobial Peptides

Antimicrobial peptides (AMPs), also known as host defense peptides, are a diverse group of small bioactive proteins (5–100 amino acids) [175] produced by almost all species as important components of their innate immune system [176]. They are classified into four structural classes: linear alpha-helical, beta-sheet, beta-hairpin or loop, and extended structures [177], although some AMPs do not fit these categories, and many only adopt their active structure upon interacting with target cell membranes [178]. It is found that many peptides form their secondary active structure only when they interact with the membranes of target cells [179].

AMPs exhibit direct microbicidal effects and indirect effects modulating inflammation to enhance cell proliferation, chemotaxis, cytokine release, angiogenesis, and wound healing. For instance, LL-37 not only kills bacteria directly but also attracts immune cells to the site of infection and promotes inflammatory cytokines release [175]. Due to their multi-faceted antimicrobial mechanisms, AMPs are seen as promising next-generation antibiotics, with a lower likelihood of resistance compared to traditional antibiotics. Thus, resistance through gene mutation is less common in AMPs, making them a more durable option in the fight against antimicrobial resistance [180].

Another key feature of AMPs is their rapid killing effect, with some being able to kill bacteria within seconds of contact with the cell membrane [176]. The presumed antimicrobial mechanisms include membrane destruction, interruption of quorum sensing, inhibition of alarm system, degrading biofilm, and inhibiting transporter expression. Many AMPs kill bacteria by disrupting membranes, causing leakage of bacterial contents. Some AMPs interact with DNA/RNA to hinder synthesis, replication, and translational processes [181]. The positive charge of AMPs enhances their binding to negatively charged prokaryotic membranes, such as those with teichoic acids in Gram-positive bacteria and LPS in Gram-negative bacteria [176]. In contrast, eukaryotic membranes have a neutral charge, reducing interaction with AMPs. Various hypothetical models explain how AMPs permeate bacterial membranes: carpet model, barrel plate model, annular pore model, aggregation channel model, and sinking raft model. According to the carpet model, peptides partition into membranes and disrupt them through electrostatic attraction to anionic phospholipids, covering the surface of the membrane in a carpet-like manner. AMPs like gramicidin, magainin, and polymyxins effectively target Gram-negative bacteria [182]. Additionally, AMPs, such as Nisin A and esculentin, have demonstrated antibiofilm activity, influencing biofilm formation and degradation through various mechanisms [183,184]. For instance, Nisin A can disrupt the membrane potential of MRSA biofilms, leading to pore formation and content leakage. Some AMPs also target stress responses in bacteria and downregulate genes involved in biofilm formation.

Despite their favorable characteristics compared to antibiotics, AMPs face several barriers to clinical use. Their in vivo antibacterial activity can be reduced by proteases and pH changes, and nonspecific interactions with cell membranes may cause host toxicity. Additionally, the high cost of solid-phase peptide synthesis (SPPS) is a concern [185]. Nonetheless, many researchers view AMPs as promising candidates for new therapies against multi-resistant infections.

### 8.4. Antibacterial Nanomaterials

Nanomaterials have shown promise in many areas of medicine due to their highly adjustable physicochemical properties, such as size, shape, and high surface-to-volume ratio, enabling them to perform significant biological activities [186,187]. They can be used in the diagnostics, prevention, and treatment of infectious diseases, which is of special importance with regard to MDR bacteria [187,188]. Besides nanomaterials used in diagnostics or for preventing bacterial attachment, there are nanoparticles with direct antibacterial activity [187]. The antibacterial effect of nanomaterials can be based on several mechanisms [186,189]. They act mostly by damaging the bacterial cell walls, membranes, or other cell components and structures through direct physicochemical interactions, or by inducing oxidative stress [186]. Their size and other properties allow them to pass through different biological barriers, including biofilms [190]. Furthermore, some types of nanoparticles can be used as drug-delivery agents, for better targeting, lowering the required dose, and reducing toxicity to the host. Polymeric nanocarriers or liposomes are most often used for loading and delivery of drugs [190]. This is especially advantageous in the treatment of intracellular bacteria [191].

As agents with active, or direct antimicrobial activity, the most promising have been the metallic and metal oxide nanoparticles, especially the widely researched silver nanoparticles [186,187]. Moreover, the use of natural compound-based nanomaterials has also been encouraged, due to their activity as inhibitors of efflux pumps or against bacterial virulence factors such as biofilm formation or motility [186]. However, the use of nanomaterials has faced several challenges. The exact effects of nanoparticles on host cells are difficult to predict, raising concerns about potential excessive oxidative stress or DNA damage [192]. Also, multiple mechanisms of nanoparticles action against bacteria make them efficient, but hard to standardize and predict the outcome. Lastly, the emergence of resistance to common nanoparticles, such as silver, has also been well documented [193].

Considering all of this, nanotechnology products have generally shown great potential, either as various antimicrobial materials themselves, or as synergistic agents with conventional antibiotics. With several of those products already FDA-approved, or in clinical trials [189], it can be expected that this approach will soon bring leverage in addressing the MDR.

### 8.5. Antibiotic Potentiators

Another approach in overcoming resistance to antibiotics is the use of antibiotic adjuvants (also called chemosensitizers [194] or resistance breakers [195])—the substances which, on their own, do not have an antimicrobial activity, but may inhibit the bacterial resistance mechanisms (Class I) and/or potentiate the effects of antibiotics when combined with them (Class II) [196], as shown in Figure 6. If used in proper ratios, the antibiotic/adjuvant combination may slow the development of resistance [197] and, importantly, due to their non-antibiotic nature, adjuvants are generally not subject to selection pressure [198]. Conversely, the main challenges are the possible safety issues (either of adjuvants themselves or when combined with antibiotics), as well as issues related to pharmacokinetics—simply put, how to achieve that both components reach the desired spatial target at about the same time in optimal concentrations [198,199].

**Figure 8:**
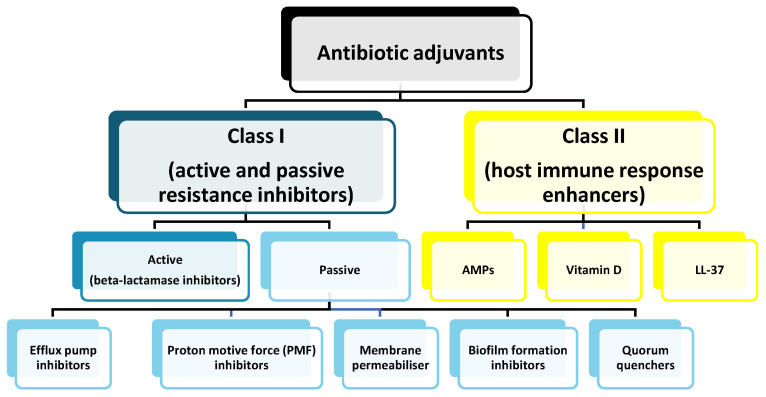
Classification of antibiotic adjuvants.

Importantly, only the active resistance inhibitors from Class I are commercially utilized, whereas the others are currently in various stages of development. Class II antibiotic adjuvants are also numerous and are described in further sections (Figure 8).

### 8.6. Host-Directed Therapy

Host-directed therapies (HDTs) are a set of therapeutic approaches that focus on improving the response of the host cells and immune system, especially in diseases characterized by the effective avoidance of these mechanisms by microorganisms [200,201]. The examples of HDTs include the use of cytokines, cellular therapy, or recombinant proteins, as well as repurposed drugs, such as metformin or aspirin [200,201]. Due to their host-directed mechanism, such therapies are less likely to cause resistance [200]. Furthermore, HDTs offer special advantages in treating intracellular bacteria specialized in avoiding host antibacterial mechanisms [200]. Tuberculosis is one of the diseases commonly addressed by using HDTs as adjuvant therapies [201,202]. Generally, HDTs are already often researched as a possible adjunctive therapy used during conventional antibiotic treatment, and it is evidently an evolving field [200].

### 8.7. Repurposed Drugs

Another option to enhance the activity of existing antibiotics is by combining them with the existing drugs indicated for non-antibiotic use, substances for which clinical development was not successful, or even those that, for various reasons have been withdrawn from the market [203]. The concept of repurposing as such is not new, with notable examples of thalidomide, sildenafil, or minoxidil. The potential advantages of such approach are reduced costs and accelerated regulatory procedures, given the fact that the safety and pharmacokinetic profile of such substances are typically well established [204].

The candidate substances for combating AMR come from such diverse groups of drugs as antidepressants (particularly serotonin reuptake inhibitors), antipsychotics (flupentixol, thioridazine, or prochlorperazine), antihypertensives (beta-blockers and calcium channel blockers, such as amlodipine, nifedipine, and verapamil), proton pump inhibitors (omeprazole, pantoprazole), NSAIDs (aspirin, diclofenac, ibuprofen, celecoxib), anesthetics (ketamine), antiparasitic (ivermectin), antidiabetics (metformin), statins (simvastatin, atorvastatin, and pitavastatin) and even antineoplastic drugs (floxuridine, mitoxantrone).

A similar diversity exists in proposed mechanisms of action that could explain laboratory data. The ongoing search is based on either existing substances or their modifications with potentially more favorable properties. Either way, it is still in the development phase despite promising initial data.

## 9. Antibiotic/Non-Antibiotic Combinations for Combating MDR Infections

AMPs are also known to enhance the activities of clinically used antibiotics through synergistic effects. Over recent decades, numerous in vitro and in vivo studies have explored combinations of AMPs with clinically used antibiotics against different pathogens.

The synergistic activity of AMPs with antibiotics against a wide range of pathogens, including MDR strains has been well documented in numerous in vitro and in vivo studies [158,205,206]. In most published studies, synergistic effects of these combinations have been shown [207,208,209]. However, some authors reported that combinations of permeabilizing cationic antimicrobial peptides and antibiotics fail to exhibit synergy [210].

Presently, nearly 6000 AMPs are listed in the data repository of antimicrobial peptides (DRAMP) with 77 in the preclinical or clinical stage of research [211]. However, only colistin (polymyxin E) and daptomycin have gained FDA approval as antibiotics. In fact, most of peptide-related antibiotics such as tyrothricin, bacitracin, and gramicidin, approved by the FDA are for topical application [212,213].

## 10. Challenges in Developing New Antimicrobials: Science, Policy, and Economics

Developing the new antimicrobial drug and making it available for worldwide use faces difficulties of various nature—scientific and technical, regulatory, and, most notably, economic/market issues.

There are numerous scientific and technical issues regarding the discovery of new active compounds with favorable physicochemical/biological properties and complexities of screening from natural sources or designing new small molecules, as well as toxicological concerns [214,215]. The latest challenges include overcoming the efflux pumps and membrane systems in MDR bacteria. Furthermore, the mode of action, interactions with other drugs, and other biochemical factors should be determined for each novel drug [214,215,216,217]. Due to the lack of newly isolated active compounds, and in order to simplify some of the technical issues, for several decades the new antibiotics have mostly been designed based on the scaffolds of previous antibiotics of synthetic or natural origin (mostly members of beta-lactams family or quinolones), consequently making it easy for bacteria to develop resistance [139,218,219]. In total, out of 13 new antibiotics approved since 2017, only 2 are representatives of new chemical classes [220].

The main reasons for the lack of new antibiotic development are, however, not of scientific, but of economic nature [214,215,216,221,222,223]. Antibiotic research in the last decades has mostly been performed in the private sector. It takes around 10 years and up to more than 2 billion dollars for a newly discovered antibacterial agent to get to the market, with chances of FDA approval being low, even for drugs that showed promise in the first phases [217,218]. The regulatory criteria are high, making it difficult to successfully complete clinical trials [224]. Moreover, even if the developers manage to complete the legislation requirements, they usually face serious losses in the market. The main reasons for this are the facts that, excluding those for MDR bacteria, other antibiotics are mostly already available and relatively cheap, and the treatment duration is usually short [221,223,224]. However, it should be noted that, despite of this, many low- and middle-income countries have limited access to effective antibiotics [225].

Having in mind the time and costs of typical research and development and subsequent multiple phases of clinical trials, as well as the stewardship protocols that limit the use of novel agents in order to postpone resistance [216,218,222], the new antibiotics generally do not bring desirable economic revenue. This results in many of the new drugs failing in the post-approval stage, not returning investment [222,223]. Being profit-oriented, most of the world’s largest pharmaceutical companies have therefore left the market of antibiotics, orienting mainly to different chronic illnesses, as can be seen from the list of FDA- and The European Medicines Agency-approved drugs in 2023 [219,226].

This left the area of research and development of antibacterial drugs to small or medium-sized enterprises (75% of companies in the field) or academia, usually funded by different “push” incentives such as grant supports, with no perspective in selling the product to bigger companies [214,223,225,227]. The projects by small companies frequently suffer from various shortfalls such as a lack of multidisciplinary teams, or the focus on narrow-spectrum agents [214]. Most significantly, small companies face difficulties in providing results of standardized toxicological studies, due to the lack of funds and short project time [214].

Lastly, even after the costly legislation process, entering the global market and commercialization remain financially burdensome, especially for drugs with limited approval for severe, resistant infections due to the small patient pool [220,223,227].

Therefore, the current lack of appropriate supportive regulative and funding mechanisms for allowing the new antibiotics to enter the market is seriously setting back the antibiotic pipeline [221,222,228].

However, the last decade has seen the rise in new incentives and policy changes aiming to improve the landscape of novel antibiotics. While the many “push” mechanisms of funding allow companies to proceed through early development phases, the supportive “pull” fundings help them in the late-stages including market entry. Some of the “pull” policies include market entry rewards, subscription models, higher reimbursement, extended exclusivity periods, etc. [216,228]. Currently active are the programs by the UK and Swedish governments for antibiotic reimbursement based on the subscription payment [216,227,228,229]. Another example of support is the Generating Antibiotic Incentives Now Act (2012) that allowed the Qualified Infectious Disease Product Designation, which accelerates the research and development, and also allows for the 5-year exclusivity of, the new drug, allowing increased revenue for sponsors [217]. Among highly promising forms of support are also the incentives to combine the public with private funding for the development of antimicrobials [215,223]. All of this brings hope that during the next decade, the most viable supportive mechanisms will be determined, allowing for the reestablishment of the functional antimicrobial development pipeline for a global supply of agents against MDR bacteria.

## 11. Biotechnology and Genomics: The Role of Biotechnology in Discovering New Antibacterial Agents, Including Genomic Approaches

Biotechnology plays a pivotal role in the discovery of new antibacterial agents, particularly in an era marked by snowballing antimicrobial resistance, with MDR pathogens becoming an increasing threat to global health. Traditional drug discovery methods often struggle to keep pace with rapidly evolving bacterial pathogens, prompting the use of innovative biotechnological approaches. One such advancement is the application of genomics, which allows researchers to investigate bacterial genomes on a large scale. Genomic sequencing [230] provides insights into bacterial resistance mechanisms and pathogenicity, enabling the identification of novel drug targets. For example, genomic approaches have led to the discovery of numerous novel antibiotics that inhibit essential bacterial processes crucial for growth and survival, such as histidine kinases (HKs), LpxC, FabI, peptide deformylase (PDF), and aminoacyl-tRNA synthetases (AaRS) [231]. Furthermore, genome-wide association studies (GWASs) have been employed to correlate specific genetic variants with antibiotic resistance profiles in bacteria [232]. This allows for the targeted design of antibacterial agents that either inhibit these resistant strains or bypass the resistance mechanisms altogether.

Another genomic approach involves the study of bacterial gene clusters responsible for the biosynthesis of natural products, many of which serve as antibacterial compounds. Genome mining can uncover these clusters in microbial genomes, enabling the discovery of new antibiotic candidates that were previously undetectable through traditional methods [233]. Moreover, techniques such as CRISPR-Cas9 have been applied to edit bacterial genomes, offering the possibility of disabling resistance genes or manipulating metabolic pathways to enhance the efficacy of existing antibiotics, or even destroying bacteria outright [234,235].

Metagenomics has also revolutionized the search for antibacterial agents. By analyzing DNA from environmental samples, researchers can discover novel microbial species and their associated antibacterial compounds without the need for culturing the organisms in a lab [236]. This approach has led to the identification of new antibiotics from previously untapped microbial sources, such as soil and marine environments, where unique bioactive compounds are often found.

Additionally, biotechnology facilitates high-throughput screening of small molecules against bacterial targets, accelerating the identification of potential drug candidates. Coupled with bioinformatics tools, these screenings can rapidly analyze vast datasets, predicting the activity and efficacy of compounds before clinical trials [237] not only shortens the time to discovery but also reduces the costs involved in the early stages of drug development.

**Figure 9:**
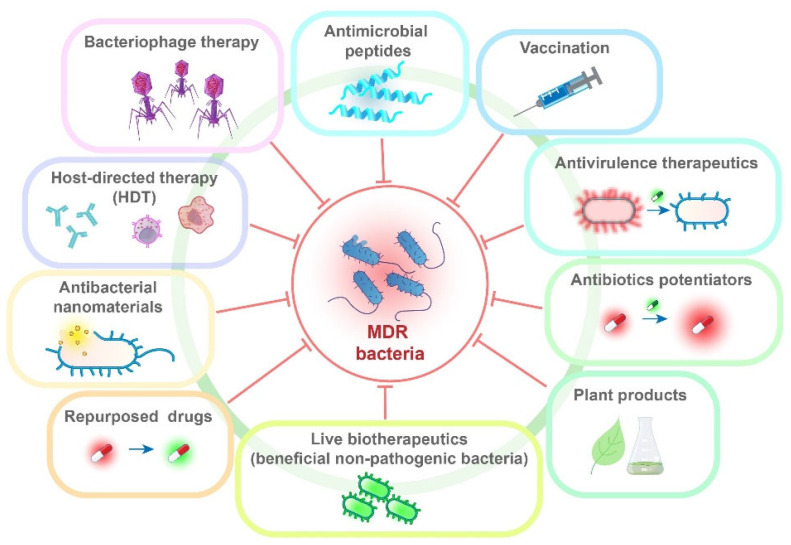
Outline of the new and currently used alternative and complementary approaches to antibiotics for combating antimicrobial resistance.

In summary, biotechnology, particularly through genomic approaches, has significantly advanced the discovery of new antibacterial agents. By leveraging the power of genomics, gene editing, and metagenomics, researchers can uncover novel drug targets, identify previously unrecognized antibiotics, and combat the growing threat of antimicrobial resistance. The overview of various approaches to combat AMR is shown in Figure 9.

## 12. Discussion

Antibacterial resistance is among the most significant global public health threats of the 21st century. This review systematically examines the burden of antibacterial resistance of the most significant pathogens and explores both existing and treatment solutions undergoing clinical and preclinical trials, providing clinicians with a clear overview of the current therapeutic landscape. While there are reviews on antibacterial agents for treating infections caused by difficult-to-treat bacteria, they primarily concentrate on specific bacterial species, genera, or groups of MDR bacteria, such as Gram-negative bacteria, carbapenem-resistant strains, or ESBL-producing bacilli. Likewise, there are reviews that focus on specific classes of antibacterial agents, such as carbapenems or BLBLI. Contrary to them, the present review offers a comprehensive analysis of all bacteria designated as “priority pathogens” by the updated report issued by the WHO [2]. It also addresses pathogens previously on the bacterial priority pathogens list (BPPL), like *C. difficile* and *H. pylori*, that remain public health threats but were excluded from the updated version. Furthermore, this review outlines available therapeutic options for significant bacterial pathogens, as well as existing management gaps. Complex issues and recent insights into infections caused by MDR bacteria are explored through a synthesis of schematic representations of fundamental concepts, including the antibiotics’ mechanisms of action and bacterial antimicrobial resistance.

One global initiative aimed at prioritizing the discovery, research, development, funding, and incentives for new antibiotics targeting drug-resistant bacterial infections led to the creation of the above-mentioned BPPL and previous ESKAPEE rankings. Given the significance of prioritizing pathogens and the increasing prevalence of antimicrobial resistance, it is crucial to develop new antibiotics and alternative or complementary antibacterial agents to combat the most challenging MDR bacteria. Since resistance to current antibiotics is inevitable, it is vital to maintain a continuous introduction of new antibacterial agents in the clinical pipeline.

Nevertheless, while the golden age of antibiotic development yielded 16 new antibiotic classes in less than 30 years, only six new classes have been launched in the subsequent five decades. The WHO recently reported that of the 32 antibiotics in development for BPPL infections, only 12 are innovative, highlighting the challenge in discovering novel antibacterials [238]. Moreover, only 4 of these 12, target at least one WHO “critical” pathogen. Furthermore, there are significant gaps throughout the pipeline, particularly concerning products for children, more convenient oral formulations for outpatients, and agents to combat increasing drug resistance [238]. An important factor in the reduced interest in antibacterial development is the current focus on chronic disease medications. Unlike long-term treatments, antibiotics are typically used for just 5 to 14 days. Furthermore, novel breakthrough antimicrobials are often reserved as last-resort options, providing insufficient economic incentive for companies. Of note, while there are antibiotics undergoing preclinical and clinical trials, most belong to the classes that are already in use. As all antibiotics belonging to an antibiotic class share the same mechanisms of action, cross-resistances quickly emerge.

However, novel classes of BLIs were approved during the past decade in different fixed drug combinations with beta-lactams, reinforcing treatment options regarding critical priority pathogens, such as carbapenem-resistant Gram-negative bacteria. Besides, new synthetic siderophore-conjugated cephalosporin, cefiderocol is active against CRAB, CRPA, and CRE.

Another promising approach seems to be the modification of existing antibiotics. For example, modifications of vancomycin have demonstrated the restoration of its efficacy against VRE [239]. Besides, findings demonstrated that combinations of certain modification approaches can enhance the efficiency even more [240].

Promisingly, non-traditional antibacterial agents such as bacteriophages, antibodies, antivirulence agents, immune modulators, products used for bacterial interference, and microbiome modulators are being increasingly investigated as alternatives or complements to antibiotics. However, studying and regulating these agents presents challenges. Aside from monoclonal antibodies targeting bacterial toxins, more efforts are needed to enhance clinical studies and evaluations to clarify the appropriate clinical uses of non-traditional antibacterials.

Alongside the discovery of new antibacterial agents, it is important to enhance methods for assessing bacterial susceptibilities to these drugs. Thus, additional research on epigenetic mechanisms and their evolving roles in antibiotic resistance should be conducted. Furthermore, current laboratory methods for assessing the susceptibility of bacteria to antimicrobials should be improved, since they are essential for managing infections and monitoring shifts in resistance trends among clinically relevant species. Standard testing often fails to mimic the complex conditions of the body, potentially resulting in inaccurate results. For example, bacteria commonly exist in biofilms, where they display different behaviors and resistance compared to planktonic cells, whereas laboratory testing methods are designed to assess the antimicrobial susceptibility of planktonic cells. Thus, current testing methods may not necessarily assess the actual antibiotic effectiveness in real-world situations.

## 13. Conclusions and Future Directions

In conclusion, to effectively address the increasing incidence of infections caused by MDR bacteria, it is crucial to complement targeted and timely antibacterial treatments with the development of new antibacterial agents, investigation of alternative approaches, use of combination therapies, and fostering interdisciplinary collaboration and global partnerships. These strategies are vital for tackling such a challenge and should be supported with policies and regulations that promote appropriate antibiotic use, strengthen infection control practices, and improve surveillance systems of emerging pathogens.

## Figures and Tables

**Table 1:** Classification of beta-lactamases, inhibitors of beta-lactamases, and their spectrum of activity. ESBL, extended-spectrum beta-lactamases; green color indicates beta-lactamase inhibitor activity; red color denotes lack of activity; carbapenemases are highlighted in red; MBL, metallo-beta-lactamases; G+, Gram-positive bacteria; G−, Gram-negative bacteria.

Beta-Lactamases	Enzyme	Active Site Residue	Host Bacteria	Target	Beta-Lactamase Inhibitor						
					2nd Generation				1st Generation		
Ambler Class					Avibactam	Enmetazobactam	Vaborbactam	Relebactam	Tazobactam	Clavulanic acid	Sulbactam
A	Narrow spectrum (SHV-1, TEM-1)	Serine-containing active site	G+ and G−	Penicillins, cephalosporins, and monobactams							
	ESBL (CTX-M, TEM-type, SHV-type)		G−	Extended-spectrum cephalosporins and monobactams (exception—carbapenems, cephamycins) [17]							
	Carbapenemase (KPC)		G−	BLAs including carbapenems							
B	Carbapenemase (NDM, IMP, VIM)	Zn-containing active site (MBLs)	G−	BLAs including carbapenems (exception—monobactams)							
C	AmpC	Serine-containing active site	Primarily G−	Penicillins, monobactams, and cephalosporins, including ceftaroline and ceftobiprole (exception—cefepime)							
D	Carbapenemase (OXA-48, other OXAs)	Serine-containing active site	Primarily G−	BLAs, including cephalosporins, cephems, and/or monobactams; some OXA-type enzymes are also capable of hydrolyzing carbapenems							

**Table 2:** Newly approved non-beta-lactam/beta-lactam inhibitor antibiotics: clinical indications and activity spectrum. G+, Gram-positive bacteria; G−, Gram-negative bacteria; CIAI, complicated intraabdominal infections; cUTI, complicated urinary tract infections; CABP, community-acquired bacterial pneumonia; SSTI, skin and soft tissue infection; BC, bacterial conjunctivitis; AOE, acute otitis externa; RTI, respiratory tract infection.

Antibacterial Agent	Antibiotic Class	Approved by FDA	Clinical Indication	Spectrum of Activity	Reference
Eravacycline	Tetracyclines	2018	CIAI, cUTIs	G+, G−, anaerobes (except *Pseudomonas aeruginosa*)	[128,129]
Omadacycline	Tetracyclines	2018	SSTI, CABP	G+, G−, anaerobes (except *P. aeruginosa*)	[130]
Plazomicin	Aminoglycosides	2018	cUTI, BSI, VAP	ESBL and CRE	[131]
Lefamulin	Pleuromutilin	2019	CABP	G+, G−, *Chlamydia trachomatis*, *Neisseria gonorrhoeae*, *Mycoplasma genitalium*	[132]
Delafloxacin	Fluoroquinolones	2017	SSTI, BC, AOE	*P. aeruginosa*, *Staphylococcus aureus*	[133]
Lascufloxacin	Fluoroquinolones	2019 (Japan)	RTI, SSTI	G+, G−	[134]
Levonadifloxacin	Fluoroquinolones	2020 (India)	RTI, SSTI	G+, G−	[134]

## Data Availability

Not applicable.

## Supplementary Materials

The following supporting information can be downloaded at: https://www.mdpi.com/article/10.3390/antibiotics14030221/s1, Table S1. Classes and subclasses of antibacterial agents, key representatives and their activity spectrum G+, Gram-positive bacteria; G−, Gram-negative bacteria; MRSA, meticillin-resistant Staphylococcus aureus; GAS, Group A streptococci; VRE, vancomycin-resistant enterococci.
