# Supplementary material for: A Comprehensive Overview of Antibacterial Agents for Combating Multidrug-Resistant Bacteria: The Current Landscape, Development, Future Opportunities, and Challenges
Source: Antibiotics (Basel). 2025 Feb 21;14(3):221. doi: 10.3390/antibiotics14030221 (PMC11939824; doi:10.3390/antibiotics14030221)
Supplement: Supplementary file 1 [file antibiotics-14-00221-s001.zip › antibiotics-3396702-Table S1.pdf]

	Class	Sub-class	Example	Spectrum of activity
Beta-lactams	Penicillins	Natural penicillins	Penicillin G, penicillin V	Beta haemolitic streptococci, *Treponema pallidum*, *Borrelia burgdorferi*
		Anti-staphylococcal penicillins (penicillinase-resistant penicillins)	Methicillin, nafcillin, oxacillin, dicloxacillin	*Staphylococcus* spp.
		Aminopenicillins	Ampicillin, amoxicillin	G+ (ear infections, UTIs, pneumonia)
		Extended-spectrum penicillins	Piperacillin, ticarcillin	*Pseudomonas aeruginosa*
	Cephalosporins	1st-generation cephalosporins	Cafazolin, cefalexin, cefadroxil, cephalotin, cefradine	G+, some G- (*Escherichia coli*)
		2nd-generation cephalosporins	Cefotetan, cefoxitin, cefuroxime, cefaclor	G+, G-
		3rd-generation cephalosporins	Ceftriaxone, cefixime, ceftibuten, ceftazidime	G+, G-, including various resistant strains
		4th-generation cephalosporins	Cefepime, cefpirome	G+, G-, including various resistant strains
		5th-generation cephalosporins	Ceftaroline, ceftobiprole, ceftolozane	G+, G-, including various resistant strains
		6th-generation cephalosporins	Cefiderocol	G-, including MBL-positive bacteria
	Carbapenems		Imipenem, meropenem, ertapenem, doripenem, panipenem, biapenem	Broadest activity: G-, including various resistant strains
	Monobactams		Aztreonam, azactam	G-, including various resistant strains
	Cyclic polypeptide		Bacitracin	Narrow spectrum of activity, only G+ (topical infections)
	N-substituted ethylenediamine		Ethambutol	*Mycobacterium* spp.
	Hydrazide derivatives		Isoniazid	*Mycobacterium* spp.
	Glycopeptides	1st-generation glycopeptide	Vancomycin, teicoplanin, ramoplanin	MRSA, *Clostridioides difficile*
		2nd-generation glycopeptide	Oritavancin, dalbavancin, telavancin	G+
	Phosphonic antibiotics		Fosfomycin	*Escherichia coli* and *Enterococcus faecalis* (cystitis) and difficult-to-treat strains such as MRSA and G-
	Polymixins		Colistin, colistimethate, polymixin B	G-
	Cyclic lipopeptides		Daptomycin	MRSA and vancomycin-resistant enterococci (VRE)
	Aminoglycosides		Gentamicin, amikacin, tobramycin, neomycin, streptomycin, plazomicin, kanamycin, spectinomycin	Aerobic G- (*Enterobacterales, Pseudomonas* spp.*), G+*
	Tetracyclines		Doxycycline, minocycline, eravacycline, tetracycline	*Chlamydia*, *Rickettsia*, mycoplasma and certain spiral bacteria, including *Borrelia burgdorferi*
		Glycylcyclines	Tigecycline	Broad spectrum of activity against both G+ (including MRSA and VRE) and G-
	Amphenicols		Chloramphenicol	Broad spectrum activity

MLS antibiotics	Macrolides		Erythromycin, clarithromycin, azithromycin	Most aerobic and anaerobic G+
	Ketolides		Telithromycin	G+
	Lincosamide		Lincomycin, pirlimycin, clindamycin	G+, anaerobic bacteria (*Bacteroides fragilis*)
	Streptogramins		Quinupristin, dalfopristin, pristinamycin, streptogramin A, B	G+, including MRSA, GAS, VRE
	Carboxylic acid		Mupirocin	G+, including staphylococci and streptococci
	Oxazolidinones		Linezolid	Resistant G+
	Fluoroquinolones	1st-generation fluoroquinolones	Nalidixic acid, cinoxacin, flumequine, oxolinic acid, piromidic acid, pipemidic acid, rosoxacin	Broad-spectrum activity
		2nd-generation fluoroquinolones	Lomefloxacin, norfloxacin, ciprofloxacin, ofloxacin, fleroxacin, pefloxacin, rufloxacin	Broad-spectrum activity, primarily targets G-, including *Pseudomonas* spp.
		3rd-generation fluoroquinolones	Levofloxacin, sparfloxacin, temafloxacin, grepafloxacin, balofloxacin, pazufloxacin, tosufloxacin	Broad-spectrum activity
		4th-generation fluoroquinolones	Moxifloxacin, gemifloxacin, trovafloxacin, gatifloxacin, clinafloxacin, garenoxacin, sitafloxacin, prulifloxacin, finafloxacin	Broad-spectrum activity, including anaerobic bacteria
	Nitrofurans		Nitrofurantoin	Common uropathogens, such as *E. coli*, *Staphylococcus saprophyticus*, *Enterococcus faecalis*
	Rifampin		Rifampin	*Staphylococcus* spp., *Mycobacterium* spp.
	Nitroimidazole		Metronidazole, tinidazole	Anaerobic bacteria
	Trimethoprim/sulfonamides		Trimethoprim/sulfamethoxazole, sulfisoxazole, sulfamethizole, sulfasalazine	Broad-spectrum activity
